# Adaptive Thermogenesis Driving Catch-Up Fat Is Associated With Increased Muscle Type 3 and Decreased Hepatic Type 1 Iodothyronine Deiodinase Activities: A Functional and Proteomic Study

**DOI:** 10.3389/fendo.2021.631176

**Published:** 2021-03-04

**Authors:** Celia Di Munno, Rosa Anna Busiello, Julie Calonne, Anna Maria Salzano, Jennifer Miles-Chan, Andrea Scaloni, Michele Ceccarelli, Pieter de Lange, Assunta Lombardi, Rosalba Senese, Federica Cioffi, Theo J. Visser, Robin P. Peeters, Abdul G. Dulloo, Elena Silvestri

**Affiliations:** ^1^ Department of Science and Technologies, University of Sannio, Benevento, Italy; ^2^ Department of Biology, Federico II University, Naples, Italy; ^3^ Department of Medicine, Physiology, University of Fribourg, Fribourg, Switzerland; ^4^ Institute for the Animal Production System in the Mediterranean Environment, National Research Council, Naples, Italy; ^5^ Department of Environmental, Biological and Pharmaceutical Sciences and Technologies, University of Campania “Luigi Vanvitelli”, Caserta, Italy; ^6^ Department of Internal Medicine, Academic Center for Thyroid Diseases, Erasmus MC, Rotterdam, Netherlands

**Keywords:** weight regain, catch-up fat, caloric restriction, thermogenesis, obesity, thrifty metabolism, thyroid hormone metabolism

## Abstract

Refeeding after caloric restriction induces weight regain and a disproportionate recovering of fat mass rather than lean mass (catch-up fat) that, in humans, associates with higher risks to develop chronic dysmetabolism. Studies in a well-established rat model of semistarvation-refeeding have reported that catch-up fat associates with hyperinsulinemia, glucose redistribution from skeletal muscle to white adipose tissue and suppressed adaptive thermogenesis sustaining a high efficiency for fat deposition. The skeletal muscle of catch-up fat animals exhibits reduced insulin-stimulated glucose utilization, mitochondrial dysfunction, delayed *in vivo* contraction-relaxation kinetics, increased proportion of slow fibers and altered local thyroid hormone metabolism, with suggestions of a role for iodothyronine deiodinases. To obtain novel insights into the skeletal muscle response during catch-up fat in this rat model, the functional proteomes of tibialis anterior and soleus muscles, harvested after 2 weeks of caloric restriction and 1 week of refeeding, were studied. Furthermore, to assess the implication of thyroid hormone metabolism in catch-up fat, circulatory thyroid hormones as well as liver type 1 (D1) and liver and skeletal muscle type 3 (D3) iodothyronine deiodinase activities were evaluated. The proteomic profiling of both skeletal muscles indicated catch-up fat-induced alterations, reflecting metabolic and contractile adjustments in soleus muscle and changes in glucose utilization and oxidative stress in tibialis anterior muscle. In response to caloric restriction, D3 activity increased in both liver and skeletal muscle, and persisted only in skeletal muscle upon refeeding. In parallel, liver D1 activity decreased during caloric restriction, and persisted during catch-up fat at a time-point when circulating levels of T4, T3 and rT3 were all restored to those of controls. Thus, during catch-up fat, a local hypothyroidism may occur in liver and skeletal muscle despite systemic euthyroidism. The resulting reduced tissue thyroid hormone bioavailability, likely D1- and D3-dependent in liver and skeletal muscle, respectively, may be part of the adaptive thermogenesis sustaining catch-up fat. These results open new perspectives in understanding the metabolic processes associated with the high efficiency of body fat recovery after caloric restriction, revealing new implications for iodothyronine deiodinases as putative biological brakes contributing in suppressed thermogenesis driving catch-up fat during weight regain.

## Introduction

The recovery of body weight after caloric restriction is often characterized by excessive adiposity, which in part results from a high efficiency of fat deposition (1–4). In an ancestral lifestyle characterized by recurrent food shortage, this physiological adaptation, also known as “catch-up fat” phenomenon, had important biological significance in optimizing the rapid restoration of the fat reserves and hence survival capacity. Nowadays, amid a modern lifestyle characterized by low physical activity and unhealthy diets, it predisposes to weight regain after therapeutic dieting, and has also been implicated in the link between the rapid fat deposition during catch-up growth and higher risks to develop chronic metabolic diseases, such as obesity, type 2 diabetes, and cardiovascular complications (5). A better understanding of the complex mechanisms underlying the thrifty metabolism driving catch-up fat may suggest novel strategies for the management of these chronic metabolic diseases.

The physiological mechanisms underlying this preferential “thrifty” metabolism are still elusive. Energy balance studies in a well-established rat model of semistarvation-refeeding have shown that the high efficiency of catch-up fat in refed animals compared to controls over a period of 2 weeks results from 10% to 15% lower energy expenditure, which is attributed to adaptive suppression of thermogenesis (3, 6). This is viewed within a hypothetical framework of a control system (referred to as an adipose-specific control thermogenesis) that operates as a feedback loop between adipose tissue depletion and skeletal muscle thermogenesis (7). Using this rat model, the suppressed thermogenesis driving catch-up fat has been shown to be accompanied by hyperinsulinemia (6) and a state of insulin resistance in skeletal muscle and insulin hyperresponsiveness in white adipose tissue (8), all of which seem to be a co-ordinated response to divert energy spared as a result of diminished glucose utilization in skeletal muscle to increased storage as fat in adipose tissues. Indeed, white adipose tissue of rats showing catch-up fat displays higher insulin-stimulated glucose utilization, increased expression of genes involved in adipogenesis and *de novo* lipogenesis, increased glucose uptake and flux towards lipid synthesis in fat stores (9, 10).

Further characterization of the skeletal muscle of rats showing catch-up fat have shown: (i) a more pronounced reduction in insulin-stimulated glucose utilization in fast-glycolytic muscles than in slow oxidative muscles (8), (ii) reduced mitochondrial mass and oxidative capacity, with a concomitant increase in ROS production despite increased ROS scavenging (11), (iii) defective PI3K, and AMPK signaling, two metabolic pathways orchestrating the substrate cycling between *de novo* lipogenesis and lipid oxidation (12), and (iv) a slower contraction and relaxation of leg muscles and increased slow fibers at the expense of fast fibers (13), all of which may contribute to the suppressed thermogenesis that drives catch-up fat.

It is well recognized that in skeletal muscle, thyroid hormones (TH) are key factors for the regulation of contractile function, metabolism, myogenesis and regeneration (14–16). Recent studies in the rat model of semistarvation-refeeding have also proposed a possible alteration of TH metabolism in skeletal muscle during catch-up fat, with a higher protein expression of the iodothyronine deiodinase type 3 (D3) and a lower protein expression of D2 in gastrocnemius muscle, and a slower net formation of triiodothyronine (T3) from exogenous thyroxine (T4) (13, 17). These findings have suggested a presumed role of iodothyronine deiodinases in the catch-up fat phenomenon, contributing to suppressed skeletal muscle thermogenesis by specifically modulating TH bioavailability and action. However, some questions still remain opened. Is the suppressed thermogenesis due to a modulation of tissue T3 disposal? Are organs other than skeletal muscle involved in impaired TH metabolism during catch-up fat? What are the biochemical and molecular pathways through which catch-up fat modulate the metabolic phenotype of skeletal muscles? Which are the pathways and the network affected in skeletal muscles during refeeding?

Here, the presumed implication of TH metabolism in the catch-up fat phenomenon has been evaluated by characterizing the TH profile, through measurements of circulating levels T4, T3, and rT3 by radio-immuno assays (RIA) and TH metabolism in peripheral target tissues, including liver and skeletal muscles, in term of D1 and D3 activities. In order to obtain novel information concerning the link between suppressed thermogenesis and insulin resistance in skeletal muscle, the protein representation profiles of tibialis anterior and soleus muscles, which differentially respond to refeeding in terms of glucose utilization and insulin sensitivity, were studied. An integrated view of the changes in TH metabolism (both at the systemic and the local level) and in the representation levels of skeletal muscle proteins, among which some T3-targets, was obtained. The overall aim of this study was to investigate the biochemical events occurring in skeletal muscles during catch-up fat upon refeeding after a period of caloric restriction. Recorded data revealed new key cellular mechanisms likely responsible for catch-up fat (i.e., altered deiodinase activities) and contribute to shed light on unknown metabolic pathways underlying the catch-up fat phenomenon, opening new perspectives for the identification of new biochemical/molecular targets for prevention and treatment of metabolic diseases.

## Materials and Methods

### Materials

All solvents used were of high performance liquid chromatography-mass spectrometry (LCMS) grade (Sigma-Aldrich, St. Louis, MO, USA and Carlo Erba, Milan, Italy). Immobilized pH-gradient (IPG) and ampholites were purchased from Bio-Rad Laboratories, Hercules, CA. Acrylamide, other reagents for polyacrylamide gel preparation, CHAPS, urea, thiourea, dithioerythriol, EDTA, iodoacetamide, brilliant blue G-colloidal concentrate, and tosyl-phenylalanyl chloromethyl ketone (TPCK)-treated porcine trypsin were from Sigma-Aldrich. ZipTip C18 micro columns were from Millipore, Bedford, MA, USA.

### Animal Model

Male Sprague Dawley rats (Elevage Janvier, Le Genest Saint Isle, France), 6 weeks of age, were acclimatized to room and cage environments for a week before the start of the experiment. The animals were caged singly and housed in a temperature-controlled room (22 ± 1°C) with a 12-h light/12-h dark cycle and maintained on chow diet (Provimi-Kliba, Cossonay, Switzerland) by energy, 24% protein, 66% carbohydrates, and 10% fat. Animals were maintained in accordance with the guidelines of the Department of Medicine (University of Fribourg) for the care and use of laboratory animals; all experimental procedures were performed under conditions approved by the Ethical Committee of the State of Fribourg Veterinary Office (Switzerland).

**Figure 1:**
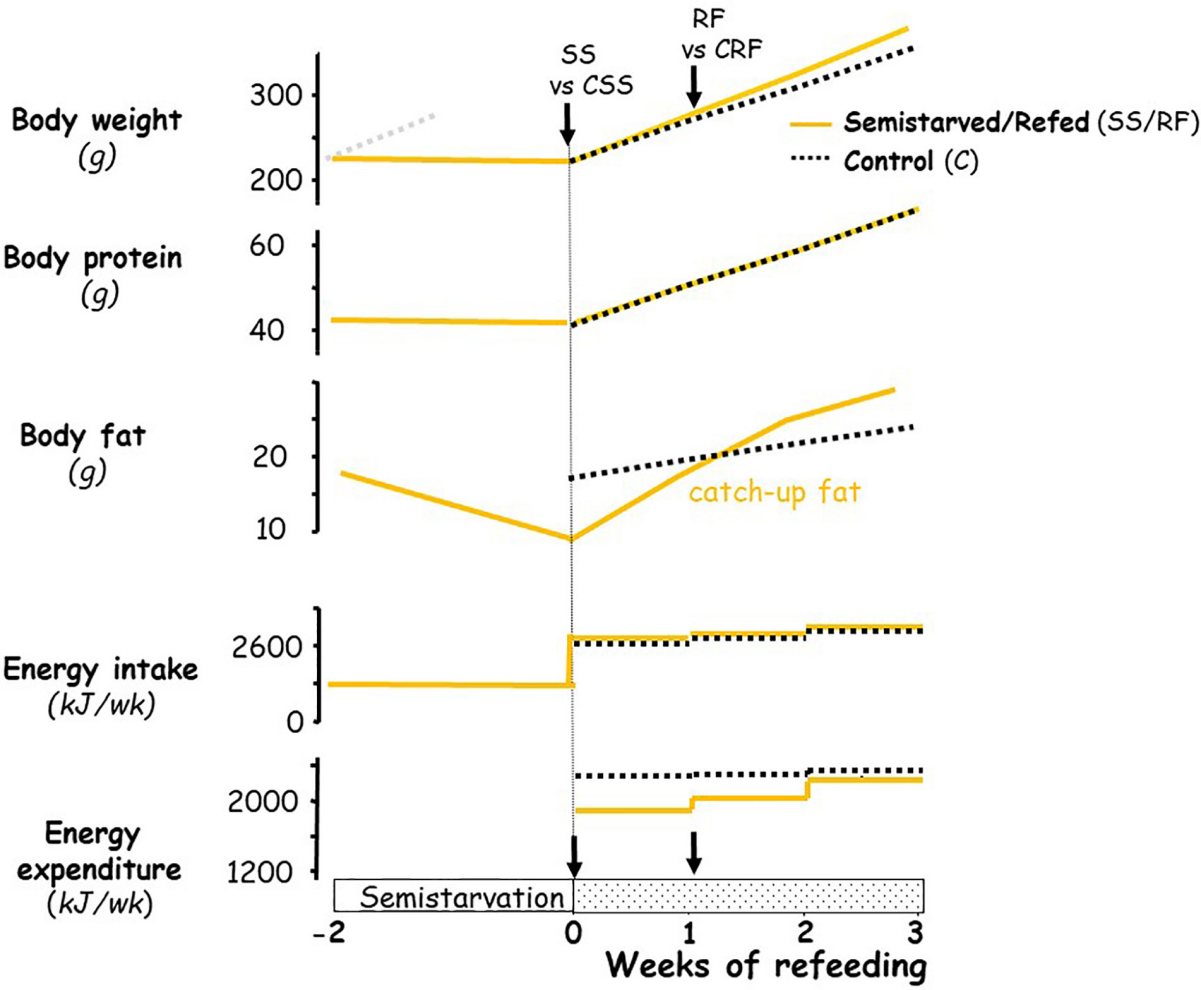
Schematic representation of the profile of body composition and energy balance in the animal model of catch-up fat during controlled refeeding after semistarvation. The schematic figure is based on data published previously on this animal model (3, 6, 18). For body weight, the faint grey dotted line represents the growth trajectory of the rats if they were not semistarved. The black arrows indicate the time-points at which tissues (and blood) were harvested, namely at the end of semi-starvation for comparison of the semi-starved (SS) animals and their respective weight-matched controls (CSS group), and after 1 week of refeeding for comparison of the refed animals (RF group) and their respective controls (CRF group).

The experimental design was similar to that previously described in establishing the rat model for studying the role of altered energy expenditure (suppressed thermogenesis) underlying the accelerated fat recovery (i.e., catch-up fat) upon refeeding after caloric restriction (3, 11, 13, 17, 18); the profile of body weight, body composition and energy balance in this rat model are depicted schematically in **Figure 1**. In brief, seven-week-old rats (225–240 g) were food-restricted at 50% of their spontaneous food intake for 2 weeks (semi-starved rats, SS); the restricted food being provided once in the afternoon (16:00–17:00) on a day-to-day basis. This level of food restriction resulted in growth arrest, i.e., without significant gain or loss in body weight and lean (protein) mass, but with about a 50% reduction in body fat relative to the onset of semi-starvation. At the end of this semi-starvation period, which corresponded to day 0 of refeeding, the semi-starved rats were refed the chow diet (the RF group) at a level approximately equal in metabolizable energy content to the spontaneous food intake of a group of rats matched for weight relative to the RF group at the onset of refeeding, and referred to as the control (CRF) group. During the phase of refeeding, the refed group therefore consumed daily the same amount of food energy as the control group fed ad libitum. Under these conditions, previous work in our laboratory has repeatedly demonstrated (depicted in **Figure 1** **)** that the refed animals showed a similar gain in lean mass, but an about 2-fold increase in body fat gain as compared to controls over a period of 2 weeks, due to 10%–15% lower energy expenditure resulting from suppressed thermogenesis. This approach allows investigations into suppressed thermogenesis specific for catch-up fat in the absence of confounding variables, such as food intake and differential rates of protein gain. A number of factors that could contribute to the difference in energetics between the refed and the weight-matched controls (namely age, locomotory activity, meal pattern) have been previously evaluated and were shown to have little or no impact on the difference in energy expenditure between the two groups (3, 6, 19, 20).

The present study was conducted on tissues harvested from SS rats and their fed controls (CSS) at the end of 2 weeks of semistarvation (time-point 0), as well as on RF rats and their weight-matched controls (CRF) after 1 week of refeeding. At these two specific time-points, all foods were removed from the cages early morning, and the rats were killed by decapitation in the early afternoon (between 13:00 and 15:00), such that all animals were most likely in the postabsorptive state. Tissues were excised, weighed, immediately frozen in liquid nitrogen and then stored at −80°C for later processing.

### Hormone Measurements

Serum T4, T3, and rT3 levels were determined by radio-immuno assay (RIA) (21). T4 antiserum was obtained from Sigma-Aldrich (St. Louis, MO), whereas T3 and rT3 antisera were produced in the Rotterdam laboratory (Erasmus University Medical Center of Rotterdam, The Netherlands). 2 × 10^4^ CPM [^125^I]-T4, [^125^I]-T3 or [^125^I]-rT3 was added for each sample. Final antibody dilutions were 1:25,000 for T4, 1:175,000 for T3 and 1:600,000 for rT3. The sample volume was 10 ml for T4, and 25 ml for T3 and rT3. The incubation mixtures were prepared in RIA buffer pH 8.6 (0.06 M barbital, 0.05 M NaOH, 0.1% BSA and 0.6 g/L 8-anilino-1-naphthalene sulfonicacid (ANS). Mixtures were incubated in duplicate overnight at 4°C and antibody-bound radioactivity complex was precipitated using Sac-Cel cellulose-coupled second antibody anti-Rabbit (IDS, Boldon, UK).

### Iodothyronine Deiodinase Activity Assays

Tissues were homogenized (POLYTRON 2500E) in 10 vol 0.1 M phosphate buffer (P100E2) pH 7.2, 2 mM EDTA and 10 mM or 20 mM dithiothreitol (DTT) for D1 or D2 and D3 activity, respectively (22). Aliquots of homogenates were stored at −80°C for further protein concentration determination. The samples were analyzed by ultra-performance liquid chromatography (UPLC) (Waters, Etten-Leur, NL) using a Waters Acquity BEH C18 reversed phase column (130 Å, 2.1 mm × 100 mm, 1.7 μm). Twenty mM ammonium acetate buffer, pH 4, (solvent A) and 0.1% acetic acid in acetonitrile (solvent B) were used as mobile phase. Flow rate, column temperature and injection volume were set on 0.35 ml min^−1^, 30°C and 100 μl, respectively. The gradient used was: 0–1 min (23% B), 1–9 min (23%–31% B), 9–15 min (31%–48% B), 15–19 min (48%–100% B) and 19–24 min (100%–23% B). The radioactivity was monitored using a Radiomatic A-500 flow scintillation detector (Packard Instruments, Meriden, CT).

### D1 Activity Assay

Liver homogenates were incubated in duplicate with 0.1 μM rT3 and 2 × 10^5^ CPM [^125^I]-rT3 in 0.1 ml P100E2 buffer and 10 mM DTT, for 30 min, at 37°C. Blank incubations were carried out in the absence of homogenate. The reactions were stopped by adding 0.125 ml of ice-cold 0.1% acetic acid in acetonitrile. The mixtures were centrifuged (1,300 x *g* for 15 min, at 4°C) and 0.1 ml of the supernatants were mixed with 0.125 ml of 20 mM ammonium acetate, pH 4, and analyzed by UPLC as previously described. The deiodinase activity of homogenates was corrected for no enzymatic deiodination observed in the blanks and for the protein concentration. D1 activity was assayed measuring the formation of radioactive 3,3′-T2 and radioiodine released from the outer ring-labeled substrate, and calculated as fmol released iodide per milligram of protein per min (fmol/mg/min). Results were expressed relative to D1 activity in controls (CSS) set as 1.

### D2 and D3 Activity Assays

For D2 activity assay, skeletal muscle (soleus, tibialis anterior, and gastrocnemius) homogenates were incubated with 1 nM T4 and 2 × 10^5^ CPM [3′-^125^I]T4 in 0.1 ml P100E2 buffer and 20 mM DTT, for 240 min at 37°C. For D3 activity assay, liver and skeletal muscle homogenates were incubated with 1 nM T3 and 2 × 10^5^ CPM [3′-^125^I]T3 for 120 or 240 min, respectively, at the same temperature and in the same buffer used for D2. Blank incubations were carried out in the absence of homogenate. The reactions were stopped by adding 0.125 ml ice-cold 0.1% acetic acid in acetonitrile. The mixtures were centrifuged (1,300 x *g* for 15 min, at 4°C), and 0.1 ml of the supernatants were mixed with 0.125 ml of 20 mM ammonium acetate (pH 4) and analyzed by UPLC as above described. The deiodinase activity of homogenates was corrected for no enzymatic deiodination observed in the blanks and for the protein concentration. D2 and D3 activities were assayed measuring the formation of radioactive T3 and 3,3′-T2 released from the outer ring-labeled substrates (i.e., T4 and T3, respectively), and calculated as fmol released iodide per milligram of protein per min (fmol/mg/min). Results were expressed relative to the enzyme activity in controls (CSS) set as 1.

### Two-Dimensional Gel Electrophoresis of Protein Extracts and Gel Image Analysis

Protein profiles of tibialis anterior and soleus muscles were studied by two-dimensional gel electrophoresis **(**2D-E), which was essentially performed as previously reported (23). In brief, frozen muscle tissue (40 mg) was homogenized in 1 ml of 8.3 M urea, 2 M thiourea, 2% CHAPS, 1% DTT, and 2% IPG buffer, pH 3–10, using a polytron. Crude extracts were vigorously shaken for 30 min, at 4°C, followed by a centrifugation at 10,000 x g, for 30 min. Then, the protein content of extracts was quantified using the Bio Rad’s DC method (Bio-Rad Laboratories, Hercules, CA). Protein extracts were prepared for each animal and assessed separately.

Samples of 650 µg of proteins were applied to immobilized pH 3-10 nonlinear-gradient strips (17 cm; BioRad). Samples of 1 mg of proteins were used for preparative gels. Focusing started at 200 V, with the voltage being gradually increased to 3,500 V and then kept constant for a further 66,500 Vh (PROTEAN IEF System; BioRad). Prior to SDS-PAGE, the IPG strips were incubated with a solution of 40 mM Tris-Cl buffer, pH 8.8, containing 6 M urea, 30%, v/v glycerol, 2%, w/v SDS, and 1%, w/v DTT, for 15 min. Strips were then equilibrated for further 15 min in the same buffer containing 5%, w/v iodoacetamide instead of DTT. The second-dimension separation was performed on 12% T SDS-polyacrylamide gels. After protein fixation, the gels were stained with colloidal Coomassie blue (Sigma-Aldrich).

Electronic images of the resulting gels were acquired by means of a calibrated densitometer (GS-800; BioRad) and analyzed using PDQuest software (BioRad). Scanned gel-images were processed for the removal of background and automatic detection of spots. For all spot-intensity calculations, normalized values were used to calculate the relative intensity (RI) for each spot: RI *= vi/vt*, where *vi* is the volume of the individual spot, and *vt* the sum of the volumes of all matched spots. For each match-set analysis, maps corresponding to protein extracts from animals of the same experimental group were organized into “Replicate Groups” (each containing 4 maps). This allowed to carry out a statistical analysis (Student’s t-test) of the experimental data relative to normalized spot densities. Spots for which the P value was less than 0.05 and with at least a 2-fold variation in pair-wise comparisons between selected experimental groups were considered to display a significant difference.

### Protein Identification by Mass Spectrometry

Spots from 2D-E were manually excised from gels, triturated and washed with water. Proteins were *in-gel* reduced, S-alkylated and digested with trypsin, as previously reported (24). Protein digests were subjected to a desalting/concentration step on µZipTipC18 pipette tips (Millipore) before nano-liquid chromatography (nLC)-electrospray ionization (ESI)-linear ion trap (LIT)-tandem (MS/MS) mass spectrometry analysis. nLC-ESI-LIT-MS/MS analysis was performed on a LTQ XL mass spectrometer (Thermo Fischer Scientific, USA) equipped with a Proxeon nanospray source connected to an Easy-nanoLC (Proxeon, Odense, Denmark). For chromatographic separation, an Easy C18 column (100 × 0.075 mm, 3 µm) (Thermo Fischer) was used at a flow rate of 300 nl/min with the following eluants: 0.1% formic acid in water (solvent A); 0.1% formic acid in acetonitrile (solvent B). Eluent gradient consisted in the following steps: 0–10 min (5%–35% B), 11–13 min (35%–95% B), 14–26 min (95% B), and 26–30 min (95%–5% B). Raw nLC-ESI-LIT-MS/MS data were searched for protein identification through MASCOT software v2.3.02 (Matrix Science, UK) using a database of *Rattus norvegicus* protein sequences from UniProtKB (10/2011, 40231 sequences). The following parameters were set for database searching: a mass tolerance value of 2 Da for precursor ion and 0.8 Da for MS/MS fragments, trypsin as a proteolytic enzyme, a missed-cleavages maximum value of 1, Cys-carbamidomethylation as fixed modification, Met-oxidation and pyro-Glu (Gln) as variable modifications. Protein candidates with more than 2 significant peptides (*P* < 0.05) with an individual MASCOT score > 30 were further evaluated by comparison with their calculated mass and pI values, using the experimental values obtained from 2D-E. Protein identity was definitively assigned if the emPAI^1st^ to emPAI^2nd^ ratio observed was >2.5 (25). Only the single protein identifications, eventually derived from the application of the above mentioned criteria, were used for the IPA analysis

The quali/quantitative analysis of the 2D-E patterns comparing tibialis anterior and soleus muscles in CCS animals allowed us to identify structural and metabolic markers differentially represented in the two muscles in control conditions (**Supplementary Data 1**).

### *In Silico* Biological Analysis

Differentially represented proteins from 2D-E were input into the IPA platform (Ingenuity^®^ Systems Ltd., USA) for the identification of functions and canonical pathways differing between the treatments. The cut-off values used were 1.5 and 0.05 for the fold change and *P*-values, respectively.

In addition, the Ingenuity Pathways Knowledge Base (IPKB) was used to analyze the whole list of differentially represented proteins in the three conditions, in terms of molecular interrelations (networks) on the basis of their connectivity. To build networks, the program utilizes the IPKB containing large numbers of individually modeled relationships between genes (obtained from the literature). The algorithm determines a statistical score for each network, by comparing the number of focus genes that contribute to a given network relative to the total number of occurrences of those genes in all networks or pathways stored in the IPKB. Then, a score is assigned to each network; the score is the negative log of *P*, and it denotes the likelihood that the focus genes in the network might be found together by chance. Therefore, scores of 2 have an at least 99% confidence of not being generated by chance alone. In addition, the biological functions assigned to each network are ranked according to the significance of that biological function to the network. A Fisher’s exact test is used to calculate *p*, indicating the probability that the assignment of the biological function to that network might be explained by chance alone.

### Statistical Analysis

Reported values are the means ± SD. All the data, except for those obtained in the 2D-E analysis (in which the used image software applies by default Student’s t-test for pairwise comparison) were evaluated by 1-way ANOVA followed by the Newman-Keuls test (GraphPad Software, La Jolla, CA, USA), with the minimum level of significance being p<0.05.

## Results

### Effects of SS and RF on TH Profile

To evaluate the thyroid state of the animals, plasma levels of T4, T3 and rT3 were measured by RIA assays in SS and RF rats and in their controls CSS and CRF, respectively.

**Figure 2:**
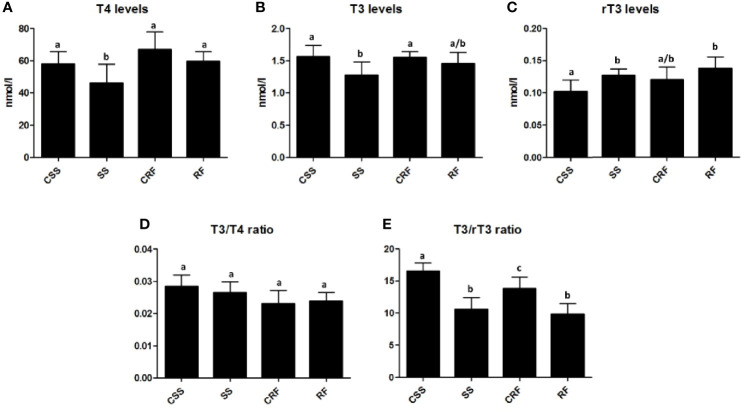
TH profile as represented by plasma levels of T4 **(A)**, T3 **(B)**, and rT3 **(C)** and resulting T3/T4 ratio **(D)** and T3/rT3 ratio **(E)** values. CSS: control of semi-starved rats; SS: semi-starved rats; CRF: control of refed rats; RF: refed rats. (**A–E**: n=4-8). Results are shown as mean values ± SD. Multiple comparisons between the groups were statistically evaluated by 1-way ANOVA followed by the Newman–Keuls test, or otherwise as indicated below. Bars labeled with dissimilar letters are significantly different; in A, SS vs. CSS and SS vs. RF, p<0.05; SS vs. CRF, p<0.001; in B, SS vs. CSS and SS vs. CRF, p<0.05; in C, RF vs. CSS, p<0.01; SS vs. CSS p<0.05, by Student’s t-test; in E, SS vs. CSS and RF vs. CSS, p<0.001; RF vs. CRF, p<0.01; SS vs. CRF and CRF vs. CSS, p<0.05.

In response to the reduced food intake, there was a significant reduction in plasma T4 (-16%) (**Figure 2A**) and T3 (-21%) (**Figure 2B**) and a concomitant significant increase in plasma rT3 (+28%) (**Figure 2C** **)** in SS (vs. CSS). In RF, the plasma levels of all of the three iodothyronines were not significantly different from those observed in CRF, indicative of a systemic euthyroidism, although rT3 showed a tendency to stay higher (+16%) (**Figures 2A–C** **)**.

To evaluate TH function and action on target tissues, the T3/T4 ratio was calculated and found not to be significantly different between the groups (**Figure 2D** **)**. On the other hand, both in SS (vs. CSS) and RF (vs. CRF), significantly reduced values were obtained for the T3/rT3 index (-37% in SS vs. CSS; -30% in RF vs. CRF); this calculated ratio reflects peripheral thyroid hormone metabolism, thus indicating a lower TH disposal in the periphery in both the nutritional conditions under study (**Figure 2E**).

### Effects of SS and RF on D1 and D3 Activities in Liver

**Figure 3:**
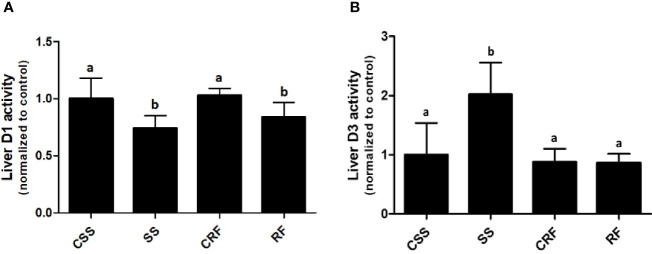
D1 **(A)** and D3 **(B)** activities in liver. CSS, control of semi-starved rats; SS, semi-starved rats; CRF, control of refed rats; RF, refed rats. D1 activity ranged from 270 to 500 fmol/min/mg. D3 activity ranged from 0.1 to 0.8 fmol/min/mg. Data were normalized to the value obtained for CSS animals (set as 1). (**A**, **B**: n= 8-10). Results are shown as mean values ± SD. Multiple comparisons between the groups were statistically evaluated by 1-way ANOVA followed by the Newman–Keuls test. Bars labeled with dissimilar letters are significantly different; in **(A)**, SS vs. CSS and SS vs. CRF, p<0.001; RF vs. CRF and RF vs. CSS, p<0.01; in **(B)**, SS vs. CSS, SS vs. CRF, and SS vs. RF, p<0.001.

To evaluate the contribution of the liver to the thyroid state of the animals as well as the local liver TH metabolism state, the hepatic D1 and D3 activities were assessed. D1 activity was significantly decreased in SS (-34%) (vs. CSS) and remained lower than controls in RF (-19%) (vs. CRF) (**Figure 3A**). D3 activity was considerably increased in SS (+100%) (vs. CSS), and returned to control levels in RF (vs. CRF) (**Figure 3B**). Thus, the liver local TH metabolism is altered in both SS and RF, prompting towards a reduced T3 bioavailability and likely a local hypothyroidism. Moreover, in SS, the lower D1 and the higher D3 activity parallel the lower T3 and the higher rT3 circulating levels. In RF, although the D3 activity is normalized, the reduced D1-dependent T4 to T3 conversion may still indicate a local hypothyroidism state that contributes to the catch-up fat phenomenon, despite the systemic euthyroid state of the animals.

### Effects of SS and RF on D3 Activity in Skeletal Muscles

**Figure 4:**
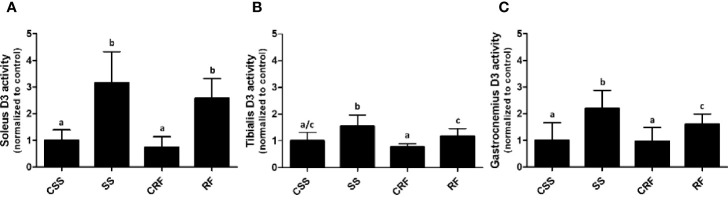
D3 activity in soleus **(A)**, tibialis anterior **(B)**, and gastrocnemius **(C)** muscles. CSS, control of semi-starved rats; SS, semi-starved rats; CRF, control of refed rats; RF, refed rats. In soleus, D3 activity ranged from 0.01 to 0.08 fmol/min/mg. In tibialis anterior, it ranged from 0.005 to 0.020 fmol/min/mg. In gastrocnemius, it ranged from 0.0013 to 0.01 fmol/min/mg. Data were normalized to the value obtained for CSS animals (set as 1). (**A, C**: n=6-10; **B**: n=5-8). Results are shown as mean values ± SD. Multiple comparisons between the groups were statistically evaluated by 1-way ANOVA followed by the Newman–Keuls test. Bars labeled with dissimilar letters are significantly different; in **(A)**, SS vs. CSS, SS vs. CRF, RF vs. CRF, and RF vs. CSS, p<0.001; in **(B)**, SS vs. CSS, p<0.01; SS vs. CRF, p<0.001, SS vs. RF, and RF vs. CRF, p<0.05; in **(C)**, SS vs. CSS and SS vs. CRF, p<0.001; SS vs. RF and RF vs CSS, p<0.05.

Analogously, to evaluate the contribution of the skeletal muscle to the thyroid state of the animals as well as the local muscle thyroid state, D2 and D3 activities were measured in soleus (**Figure 4A**), tibialis anterior (**Figure 4B**), and gastrocnemius (**Figure 4C**).

In all of the three muscles, while D2 activity was undetectable (data not shown), D3 activity was significantly increased in SS (vs. CSS) (+200%, +50%, +116%, in soleus, tibialis anterior, and gastrocnemius, respectively) and remained higher than controls in RF (vs. CRF) (+260%, +50%, +70%, in soleus, tibialis anterior, and gastrocnemius, respectively) (**Figures 4A–C** **)**. As D3 catalyzes the inactivation of T4 to rT3, the data during RF indicates a sustained alteration of TH metabolism also in skeletal muscle and specifically a D3-dependent reduced bioavailability of T3 likely corresponding to a local hypothyroidism, which could be an additional mechanism putatively contributing to the catch-up fat phenomenon. Moreover, the higher D3 activity in skeletal muscle further explains the higher rT3 plasma levels observed in SS, and tendentially in RF.

### Effects of SS and RF on Protein Profile of Tibialis Anterior and Soleus Muscles

With the aim to gain insights into the effects exerted by SS and RF on the skeletal muscle phenotypes, solubilized proteins from tibialis anterior and soleus (i.e., two muscles, previously studied for D3 activity, showing quite opposite metabolic phenotype, prevalently fast-glycolytic and slow-oxidative, respectively) of CSS, SS, CRF, and RF rats were subjected to a 2D-E-based proteomic analysis.

**Figure 5:**
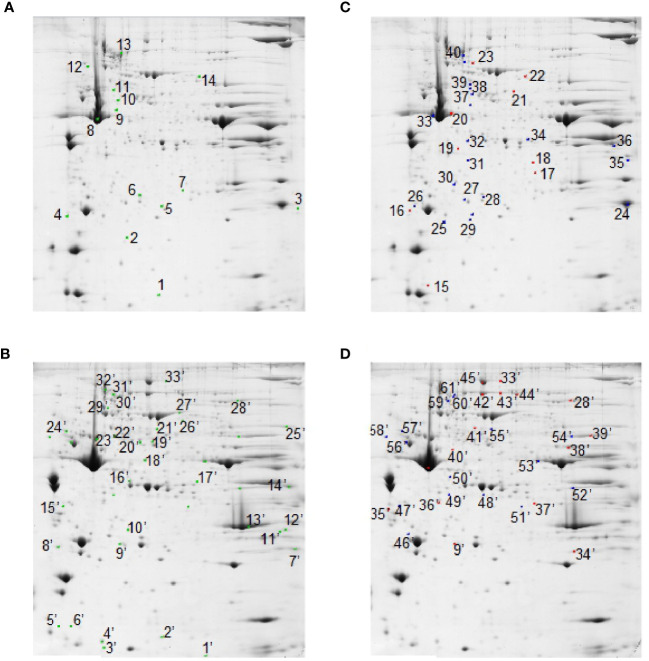
2D-E based quantitative analysis of skeletal muscle proteomes. Representative 2D-E Coomassie Blue stained maps of total soluble proteins from tibialis anterior **(A, C)** and soleus **(B, D)**. 2D-E was performed using a nonlinear pH range of 3–10 in the first dimension (17 cm strips) and SDS-PAGE (12% T) in the second dimension. Protein loading was 650 µg/gel. Green crosses show differentially represented spots (p<0.05-at least 2-fold change) in the SS vs. CSS comparison in tibialis anterior **(A)** and soleus **(B)**. Red crosses show differentially represented spots (p<0.05-at least 2-fold change) in the comparison RF vs. SS in tibialis anterior **(C)** and soleus **(D)**. Blue crosses show differentially represented spots (p<0.05-at least 2-fold change) in the RF vs. CRF comparison in tibialis anterior **(C)** and soleus **(D)**. Statistical significance in pair-wise comparisons was evaluated by Student’s t-test.

At the detection-limits set, image software counted 350 common matched proteins among the various electrophoretic maps in both muscles. Pair-wise comparisons were performed to analyze the differential representation pattern associated with SS and RF. Limiting our interest to a differential representation of at least 2-fold and a statistical significance of at least 95% (p<0.05), 14 (about 4% of total entries) and 33 spots (about 10% of total entries) resulted in the detection of significant quantitative changes in SS vs. CSS, in tibialis anterior and soleus muscles, respectively (**Figures 5A, B**, green spots). Upon RF, 9 (3% of total entries) and 16 (5% of total entries) differentially represented spots (p< 0.05- at least 2-fold change) were detected in the comparison RF vs. SS in tibialis anterior and soleus, respectively (**Figures 5C, D**, red spots). 21 and 16 spots resulted to be differentially represented in the comparison between RF vs. CRF in tibialis and soleus, respectively (**Figures 5C, D**, blue spots).

Among the 14 differentially represented proteins in tibialis anterior upon SS, 3 (21%) were over-represented and 11 (79%) were down-represented vs. CSS. Among the nine differentially represented proteins in tibialis anterior upon RF, three (33%) were over-represented and six (67%) were down-represented vs. SS. Among the 33 differentially represented proteins in soleus upon SS, 12 (36%) were over-represented and 21 (64%) were down-represented vs. CSS. Among the 16 differentially represented proteins in soleus upon RF, eight (50%) were over-represented and eight (50%) were down-represented vs. SS. These quantitative proteomic data suggested that the two muscles presented a differential proteomic flexibility, with tibialis anterior showing vs. soleus about half of altered proteins in both SS (vs. CSS) and RF (vs. SS), and a quite different pattern of over- and down- representation in RF.

Nano-LC-ESI-LIT-MS/MS analysis allowed the assignment of 6, 7, and 11 differentially represented proteins in tibialis anterior upon SS vs. CSS, RF vs. SS, and RF vs. CRF, respectively. On the other hand, it demonstrated the occurrence of 20, 6, and 10 differentially represented proteins in soleus, in the same pair-wise comparisons, the remaining spots being not processed due to their low quality or quantity (for identification details, see **Supplementary Data 2**).

**Figure 6:**
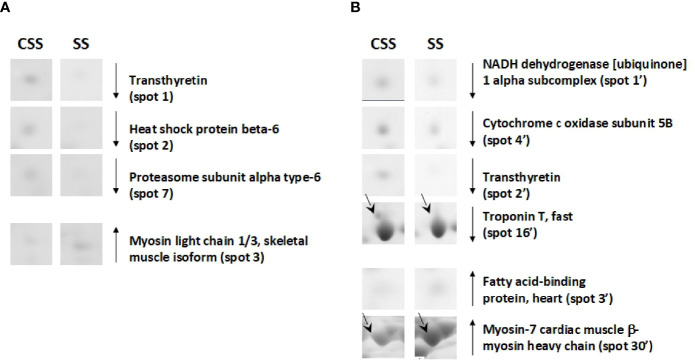
SS–dependent differentially represented proteins in tibialis anterior **(A)** and soleus **(B)**. Representative subsections of 2D-E images of tibialis anterior and soleus. CSS, control of semi-starved rats; SS, semi-starved rats. A selection of spots is shown. Identified protein spots belong to those showing a relative intensity that differed significantly between experimental groups by at least 2-fold with a p<0.05 (Student’s t-test). Down arrows mean reduced representation; up arrows mean increased representation. Effects of SS are reported vs. CSS. Spot numbering refers to **Figure 5** and **Supplementary Data 2**.

Based on 2D-E data, it was evident that the SS condition produces (vs. CSS), both in tibialis anterior and in soleus, a proteomic modulation that was mainly associated with a reduction of the protein representation levels. When compared to that of CSS counterparts, tibialis anterior of SS rats showed reduced representation levels of proteins such as heat shock protein beta-6 (spot 2) and proteasome subunit alpha type-6 (spot 7) (**Figure 6A** **)**. When compared to that of controls, soleus of SS rats showed reduced representation levels of proteins such as troponin T, fast (spot 16’) and NADH dehydrogenase [ubiquinone] 1 alpha subcomplex (spot 1’) (**Figure 6B**). In the SS condition, both tibialis anterior and soleus showed reduced representation levels of transthyretin (spot 1 in tibialis anterior; spot 2’ in soleus) (**Figures 6A, B** **)**. At the same time, both muscles were characterized by increased representation levels of isoforms of contractile proteins indicative of a slow/oxidative phenotype, such as myosin light chain 1/3 (spot 3) in tibialis anterior, and myosin-7 cardiac (spot 30’ and 31’) in soleus (**Figure 6**).

**Figure 7:**
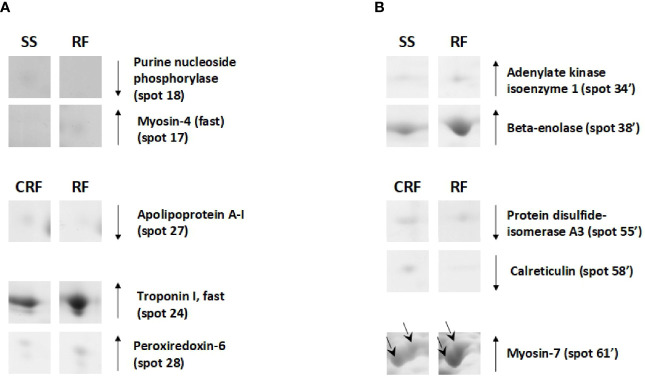
RF–dependent differentially represented proteins in tibialis anterior **(A)** and soleus **(B)**. Representative subsections of 2D-E images of tibialis anterior and soleus. SS, semi-starved rats; CRF, control of refed rats; RF, refed rats. A selection of spots is shown. Identified protein spots belong to those showing a relative intensity that differed significantly between experimental groups by at least 2-fold with a p<0.05 (Student’s t-test). Down arrows mean a reduced representation; up arrows mean an increased representation. Effects of RF are reported vs. SS and CRF. Spot numbering refers to  **Figure 5** and **Supplementary Data 2**.

On the other hand, the RF condition also generally produced a reduction of the protein representation levels in tibialis anterior; this was not the case for soleus. In particular, when compared to that of SS counterparts, tibialis anterior of RF rats showed reduced representation levels of proteins such as purine nucleoside phosphorylase (spot 18) and increased representation levels of proteins among which myosin-4 fast (spot 15), which is a molecular marker of fast fibers (**Figure 7A**). Moreover, in comparison to that of CRF counterparts, tibialis anterior of RF rats showed a reduced representation levels of apolipoprotein A-I (spot 27) that is likely indicative of reduced glucose utilization, and increased representation levels of peroxiredoxin-6 (PRDX-6) (spot 28) and troponin I, fast (spot 24), which are molecular markers of augmented oxidative stress and fast fibers, respectively (**Figure 7A**).

In soleus, the RF condition produced mainly representation level changes of key metabolic enzymes (**Figure 7B** **)**. When compared to SS rats, RF ones showed increased representation levels of enzymes such as adenylate kinase isoenzyme 1 (spot 34’) and beta-enolase (spot 38’) (**Figure 7B** **).** When compared to CRF rats, RF ones showed reduced representation levels of stress enzymes such as protein disulfide-isomerase A3 (spot 55’) and calreticulin (spot 58’), and increased representation levels of proteins such as myosin-7 (spot 60’ and 61’), which is a molecular marker of slow fibers (**Figure 7B** **)**.

### *In Silico* Analysis of Differential Represented Proteins in Tibialis Anterior and Soleus Muscles

**Figure 8:**
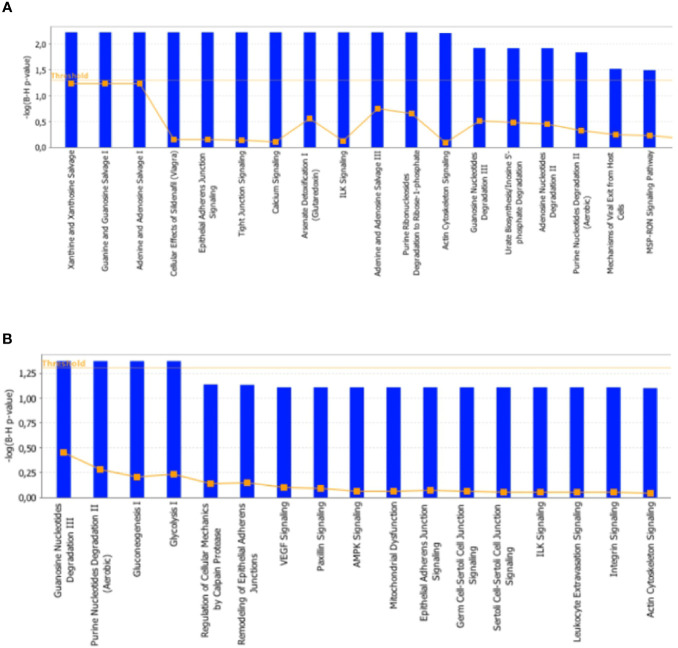
*In silico* analysis of differentially represented proteins upon refeeding; canonical pathways affected in tibialis anterior **(A)** and soleus **(B)** muscles upon RF vs. SS comparison. The lists of differentially represented proteins were input into the IPA platform (Ingenuity® Systems, www.ingenuity.com) for the identification of canonical pathways. The results, corresponding to the value of the fold change between SS and RF, are reported. The cutoff was 1.5 for the fold change and a p-value of 0.05.

To further characterize the effects elicited by the RF condition in rat skeletal muscles, proteomic data were *in silico* analyzed by using the IPA platform that, based on known interactions between affected proteins, defines common functional and canonical pathways as well as protein networks, thereby offering additional information about the interactive links between modulated proteins following the treatments under study. When the interest was limited to the effect exerted by RF vs. SS, the analysis of the pathways showed alterations involving, among the others, calcium and ubiquitination signaling in tibialis anterior (**Figure 8A** **),** and glucose metabolism (gluconeogenesis, glycolysis, AMPK signaling), mitochondrial dysfunction, and proteolysis (regulation of cellular mechanisms by calpain protease) in soleus (**Figure 8B**).

**Figure 9:**
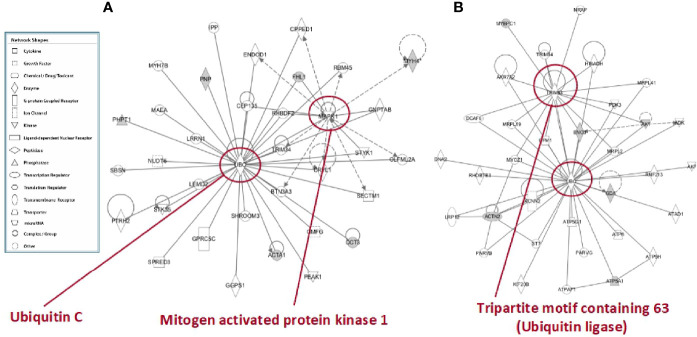
*In silico* analysis of interconnections between differentially represented proteins in tibialis anterior **(A)** and soleus **(B)** muscles upon RF vs. SS comparison. Gene products are represented as nodes, and the biological relationship between two nodes is represented as an edge (line). Indirect interactions appear as broken lines, whereas direct interactions appear as solid lines. All edges shown are supported by at least 1 reference from the literature, from a textbook, or from canonical information stored in the Ingenuity Pathways Knowledge Base (IPKB). Human, mouse, and rat orthologues of a gene are stored as separate objects in IPKB but are represented as a single node in the network. For clarity, network shapes are shown. In A: ACTA1, actin, alpha skeletal muscle; BTN3A3, utyrophilin subfamily 3 member A3; CCT3, T-complex protein 1 subunit gamma; CEP135, centrosomal protein 135; CPPED1, serine/threonine-protein phosphatase; CRYL1, lambda-crystallin homolog; ENDOD1, endonuclease domain containing protein 1; GGPS1, geranylgeranyl pyrophosphate synthase; GMFG, glia maturation factor gamma; GNPTAB, N-acetylglucosamine-1-phosphate transferase subunits alpha and beta; GPRC5C, G-protein coupled receptor family C group 5 member C; IPP, actin-binding protein IPP; LEMD2, LEM domain containing 2; LRRN1, leucine-rich repeat neuronal protein 1; MAEA, E3 ubiquitin-protein transferase MAEA; MYH4, myosin heavy chain 2. MYH7B, myosin heavy chain 7B; NUDT6, nucleoside diphosphate-linked moiety X motif 6; OLFML2A, olfactomedin-like 2A; PEAK1, pseudopodium-enriched atypical kinase 1; PHPT1, 14 kDa phosphohistidine phosphatase; PNP, purine nucleoside phosphorylase; PTRH2, peptidyl-tRNA hydrolase 2; RBM45, RNA-binding protein 45; RHBDF2, rhomboid 5 homolog 2; SBSN, suprabasin; SECTM1, secreted and transmembrane 1; SHROOM3, shroom family member 3; SPRED3, sprouty-related, EVH1 domain-containing 3; STK35, serine/threonine kinase 35; STYK1, serine/threonine/tyrosine kinase 1; TRIM34, tripartite motif-containing 34; UBC, ubiquintin **(C)** In B: ACTN2, actinin alpha 2; ADK, adenosine kinase; AK1, adenylate kinase isoenzyme 1; AK7, adenylate kinase 7; AKR7A2, aflatoxin B1 aldehyde reductase member 2; ATP5A1, ATP synthase, mitochondrial F1 complex, alpha subunit 1; ATP5G1, ATP synthase F(0) complex subunit C1, mitochondrial; ATP5H, ATP synthase subunit d, mitochondrial; ATP6, ATP synthase subunit a; ATPAF1, ATP synthase mitochondrial F1 complex assembly factor 1; ATPD1, ATPase family AAA domain-containing protein 1; DCAF6, DDB1 and CUL4-associated factor 6; DNA2, DNA replication ATP-dependent helicase/nuclease; ENO3, beta-enolase; GDA, guanine deaminase; GFM1, elongation factor G, mitochondrial; HIBADH, 3-hydroxyisobutyrate dehydrogenase, mitochondrial; KCNN2, small conductance calcium-activated potassium channel protein 2; KIF20B, kinesin family member 20B; LRP12, LDL receptor-related protein 12; MRPL19, mitochondrial ribosomal protein L19; MRPL2, 39S ribosomal protein L2, mitochondrial; MRPL2, 39S ribosomal protein L2, mitochondrial; MRPL41, 39S ribosomal protein L41, mitochondrial; MYBPC1, myosin-binding protein C, slow-type; MYOZ1, myozenin 1; NRAP, nebulin-related-anchoring protein; PARVB, parvb protein; PARVG, parvin; PDK3, protein-serine/threonine kinase; RHOBTB3, Rho-related BTB domain containing 3 (predicted), isoform CRA_b; RNF213, ring finger protein 213; ST7, suppressor of tumorigenicity 7 protein; TRIM54, tripartite motif-containing protein 54; TRIM63, E3 ubiquitin-protein ligase; UBC, ubiquintin C.

In tibialis anterior, the network analysis produced inter-connections among the differentially represented proteins (RF vs. SS) around two protein nodes, namely ubiquitin C (UBC) and mitogen-activated protein kinase 1 (MAPK1) (**Figure 9A**). In soleus, the same network analysis (RF vs. SS) highlighted UBC and Tripartite motif containing 63 (TRIM63) as high score nodes (**Figure 9B** **)**. UBC, the node in common between the networks of the two muscles, is the product of one of the four genes coding ubiquitin in mammals; recently, it has been described as the most sensible gene product in response to heat and oxidative stress (26). As far as it concerns MAPK1, this protein plays a pivotal role in the MAPK/ERK pathways affecting diverse biological functions including, among others, cell survival and cytoskeleton rearrangement. Interestingly, alterations in the MAPK pathways have been shown to represent the molecular signatures associated with skeletal muscle insulin resistance (27)*;* the latter condition, upon RF, is a feature of tibialis anterior and not of soleus. Finally, TRIM63 is a ubiquitin ligase selectively expressed in striated muscles and elicits a central role in maintaining protein homeostasis in these tissues (28). The presence of the UBC node in both networks and the concomitant presence of the TRIM63 node in the soleus network strongly suggest that ubiquitin-dependent cellular functions may have a central role in the metabolic and structural adaptations occurring, upon RF, both in tibialis and in soleus, likely exacerbated in soleus.

## Discussion

In this study, we have focused on skeletal muscle TH metabolism during catch-up fat. In line with the results reported in (18), and coherently with the literature concerning TH economy in caloric restriction in humans and rodents (29, 30), plasma T4 and T3 levels were decreased at the end of SS (SS vs. CSS); however, upon RF, plasma T4 and T3 were restored to control levels (RF vs. CRF). On the other hand, SS, and tendentially RF, produced higher levels of plasma rT3 (SS vs. CSS), suggesting an increased inactivation of T4 to rT3, thus supporting the hypothesis of lower T3 availability in peripheral tissues in both nutritional conditions. Further support for this hypothesis can be derived from the observed reduction of the index reflecting the peripheral TH metabolism, namely the T3/rT3 ratio (31), reduced in SS and persisting lower upon refeeding. All this can be part of a feedback loop phenomenon with adipose tissue fat depletion and repletion.

The control of iodothyronine plasma levels correlates with the peripheral conversion of T4 by iodothyronine deiodinase enzymes; specifically, an altered coordination of deiodinases in tissues, meant as reduced activation of T4 to T3 by D1/D2 enzymes and high inactivation of T4 to rT3 by D3, decreases circulating T3 levels (32). In addition, the regulation of deiodinase expression and activity in metabolically relevant tissues, also determine the cell-specific thyroid state and the intracellular TH activity, an effect that participate in the systemic modulation of metabolism and energy expenditure (33), even independently of the circulating TH levels.

In our hands, at the liver level, SS reduced D1 activity (i.e., reduced T4 to T3 conversion) and increased that of D3 (i.e., increased T4 inactivation to rT3), strictly in parallel to changes in plasma T3 and rT3 levels. Previous studies reported, under caloric restriction, a direct relation between TH levels and D1/D3 activity/expression in liver (34–37). On the other hand, after the re-establishment of the normal food intake, liver activity of D3 but not that of D1, was restored at control values (RF vs. CRF), D1 activity persisting at levels below those of controls, despite the systemic euthyroid state of the refed animals. Thus, a “conserved” suppression of liver D1 activity in RF upon SS and the putative persistence of a D1-dependent diminished local T3 bioavailability and action, i.e., a liver local hypothyroidism, might contribute to the catch-up fat phenomenon.

As far as the skeletal muscle is concerned, the intra-cellular levels of T3 are the result of the coordinated actions of D2 and D3 (16). In all investigated muscles, D3 activity was strongly induced upon SS, and such a condition persisted upon refeeding, suggesting (even in absence of information on D2 activity) a likely local skeletal muscle hypothyroidism, despite resumption of normal food intake and the systemic euthyroid state of the refed animals. Recorded data are coherent with findings of decreased rate of muscular T3 synthesis and changes in the protein levels of deiodinases (13, 17). Moreover, it is known that caloric restriction induced hypothyroidism produces in skeletal muscle a fast-to-slow fiber transition, which was well documented in hypothyroid patients, chemically induced hypothyroid rodents, or THR-deficient mice (14, 23, 38). Consistent with the hypothesis of an RF-induced skeletal muscle hypothyroidism are the findings that the skeletal muscle of refed rats are characterized by *in vivo* delayed contraction-relaxation rate and higher ratio of slow to fast fibers (13). Our data further support an RF-induced imbalance of T3 metabolism in skeletal muscle, showing a role for D3 in the prevalence of T4 inactivation to rT3. The putative contribution of regulatory mechanisms concerning D2 activity requires further analysis. Indeed, the role of D2 activity during weight regain in skeletal muscles as well as in other tissues, such as adipose tissues, still remains elusive.

To further characterize the fiber type-specific response to SS and RF, the corresponding proteomic profiles of tibialis anterior and soleus muscles were analyzed. Noteworthy, in SS, in both muscles, our results indicate a significant down-representation of transthyretin (TTR), the transporter protein for T_4_ in the blood (39–42). The liver is considered the main contributing organ for TTR release, but a recent study has shown TTR synthesis in muscle cells, where the protein has been suggested to exocytose through exosomes and bring back T_4_ inside the cells for conversion to T3 (43). The reduced representation levels of TTR in tibialis anterior and soleus of SS animals appear coherent with a locally reduced T3 bioavailability. Moreover, SS condition was associated in both muscles with increased representation levels of slow myosin isoforms, namely myosin light chain 1/3 in tibialis anterior, and myosin 7 in soleus, the latter directly regulated by T3 through a negative TRE (44), confirming in SS reduced T3 disposal in skeletal muscle and a shift towards slow fibers. In soleus, such a shift is further supported by reduced representation levels of troponin T fast. A local muscle hypothyroidism could also produce a depressed mitochondrial biogenesis (45). In soleus, the reduced representation levels of NADH dehydrogenase (ubiquinone) 1α subcomplex and cytochrome c subunit V, support such idea.

On the other hand, RF likely produces different effects in the two analyzed muscle. In tibialis anterior, a shift back towards a fast phenotype was suggested by the observed increased representative levels of myosin-4 fast and troponin I fast. In soleus, metabolic adjustments were detected, as deduced by the increased representative levels of β-enolase, adenylate kinase isoenzyme 1, and ATPase α-subunit, together with the persistence of the SS-induced contractile features, such as increased representation levels of slow myosin isoforms (i.e., myosin 7). In tibialis anterior, the RF-restored fast phenotype is also associated with reduced levels of apolipoprotein A-I, and increased representation of peroxiredoxin-6, likely indicative of reduced glucose utilization (46) and higher free radical scavenging activity (47), respectively. Such features appears in line with the previously reported RF-induced reduced glucose utilization index in tibialis anterior (8).

*In silico* analysis confirmed alterations in glucose and lipid metabolism induced by caloric restriction and refeeding. Differential pathways analysis (RF vs. SS) reported, among the others, alterations in calcium and structural signaling in tibialis, and in glycolysis and gluconeogenesis pathways, AMPK signaling and mitochondrial dysfunction in soleus. Differential network analysis identified the central presence of the UBC node in both tibialis anterior and soleus muscle. UBC is the most responsive gene to several types of stresses, e.g., starvation (48–51). It acts by increasing the ubiquitin supply needed to counteract both proteotoxic and genotoxic stress (26, 52), and has been suggested to be involved in whole-body energy balance (53, 54). For tibialis anterior, another central network node was MAPK1 (or p38MAPK), whose pathway is essential for glucose homeostasis and insulin signaling (55), and is induced by mitochondrial dysfunction, ROS damage and inflammation (56–58). In line with the present network analysis, previous studies reported in skeletal muscle of refed rats: i) increased mitochondrial ROS production and antioxidant defenses as well as diminished mitochondrial mass and oxidative capacity (11); ii) lower PI3K activity (basal and *in vivo* insulin-stimulated) and impaired AMPK phosphorylation (basal and *in vivo* leptin-stimulated) (12). Of note, upon RF, insulin resistance is a feature of tibialis anterior and not of soleus. In soleus, the other central network node was TRIM63, an E3 ubiquitin ligase (59), contributing to the clearance of the misfolded proteins as well as to muscle protein homeostasis (28). These *in silico* data suggest ubiquitination pathways and protein turnover as central events in the adaptation occurring in muscle upon RF. Coherently, a recent study has reported a concomitant reduction in both protein synthesis and protein degradation rates in skeletal muscles of refed animals (17). This highlighted protein turnover, an energetically costly “futile” cycle, as an additional “suppressed pathway” likely driving the thrifty metabolism, which rapidly restores the fat reserves during weight regain after caloric restriction.

It should be noted that the interpretation of data, when comparing responses of caloric restricted animal and their ad libitum fed controls, is complicated by between-group differences in feeding behaviour and pattern. In particular a more gorging behavior in caloric restricted animals is observed when food is given once and consumed within a few hours (as in our study). Clearly, this could be a factor in explaining some of the observed differences at the end of the semi-starvation period, when comparing the SS and CSS groups. However, since in our rat model the gorging behavior disappears within the first few days of refeeding, the impact of differential feeding pattern on our comparisons of RF and CRF groups is unlikely to be of major importance after 7 days of refeeding, i.e., a time-point when the gorging behaviour would have subsided, and which reflects the mid-point of the dynamic phase of catch-up fat.

Overall, the characterization of the TH peripheral metabolism together with the proteomic data demonstrate that a tissue specific modulation of deiodinase activities may be one of the mechanisms underlying the suppressed thermogenesis driving catch-up fat. Refeeding after caloric restriction affects differential pathways in glycolytic and oxidative muscles, which involve contractile protein isoforms, metabolic enzymes and components involved in response to oxidative stress and converge on factors involved in protein turnover. Specifically, iodothyronine deiodinase enzymes appear as putative biological brakes able in reducing T4 activation (D1) (in liver) or increasing its inactivation (D3) (in skeletal muscles), thus acting as peripheral mediators of suppressed thermogenesis driving catch-up fat, independently of the systemic thyroid state of the animals. In perspective, a hypothetical D3 skeletal muscle-specific modulation by diet, nutraceuticals, and pharmacological approaches might represent a novel target to counter the local hypothyroidism and hence enhance skeletal muscle (and whole-body) thermogenesis for countering catch-up fat and obesity.

## Data Availability Statement

The raw data supporting the conclusions of this article will be made available by the authors, without undue reservation.

## Ethics Statement

Animals were maintained in accordance with the guidelines of the Department of Medicine (University of Fribourg) for the care and use of laboratory animals. All experimental procedures were performed under conditions approved by the ethical committee of the State of Fribourg Veterinary Office (Switzerland).

## Author Contributions

CD performed TH measurements, deiodinase activity assays, elaborated data, and wrote and revised the manuscript. RB performed proteomic analyses, elaborated data, and revised the manuscript. AMS performed MS analyses and revised the manuscript. AS supervised MS data elaboration and revised the manuscript. JC and JM-C supervised animal care and treatments, and revised the manuscript. FC contributed to design the experimental approaches and revised the manuscript. MC performed *in silico* analyses and revised the manuscript. PD, AL, and RS contributed to design the experimental approaches and revised the manuscript. RP and TV supervised TH measurements and deiodinase activity assays and revised the manuscript. AD designed the experimental model and approaches, supervised animal care and treatments, and revised the manuscript. ES performed proteomic analyses, supervised data elaboration, and wrote and revised the manuscript. All authors contributed to the article and approved the submitted version.

## Funding

This research was funded by the University of Sannio Research Grants and in part by the Swiss National Science Foundation (grant 310030-152870).

## Conflict of Interest

The authors declare that the research was conducted in the absence of any commercial or financial relationships that could be construed as a potential conflict of interest.

## References

[1] Keys A Brozek J Henschel A Mickelson O Taylor HL. The Biology of Human Starvation. Minneapolis, MN: University of Minnesota Press (1950).

[2] Boyle PC Storlein LH Keesey RE. Increased efficiency of food utilization following weight loss. Physiol Behav (1978) 21:261–4. 10.1016/0031-9384(78)90050-1 693652

[3] Dulloo AG Girardier L. Adaptive changes in energy expenditure during refeeding following low-calorie intake: evidence for a specific metabolic component favoring fat storage. Am J Clin Nutr (1990) 52(3):415–20. 10.1093/ajcn/52.3.415 2393003

[4] MacLean PS Higgins JA Johnson GC Fleming-Elder BK Donahoo WT Melanson EL. Enhanced metabolic efficiency contributes to weight regain after weight loss in obesity-prone rats. Am J Physiol Regul Integr Comp Physiol (2004) 287:R1306–15. 10.1152/ajpregu.00463.2004 15331386

[5] Dulloo AG Jacquet J Seydoux J Montani JP. The thrifty ‘catch-up fat’ phenotype: its impact on insulin sensitivity during growth trajectories to obesity and metabolic syndrome. Int J Obes (Lond) (2006) 30(Suppl 4):S23–35. 10.1038/sj.ijo.0803516 17133232

[6] Crescenzo R Samec S Antic V Rohner-Jeanrenaud F Seydoux J Montani JP. A role for suppressed thermogenesis favoring catch-up fat in the pathophysiology of catch-up growth. Diabetes (2003) 52:1090–7. 10.2337/diabetes.52.5.1090 12716737

[7] Dulloo AG Jacquet J. An adipose-specific control of thermogenesis in body weight regulation. Int J Obes Relat Metab Disord (2001) 5:S22–9. 10.1038/sj.ijo.0801907 11840210

[8] Cettour-Rose P Samec S Russell AP Summermatter S Mainieri D Carrillo-Theander C. Redistribution of glucose from skeletal muscle to adipose tissue during catch-up fat: a link between catch-up growth and later metabolic syndrome. Diabetes (2005) 54(3):751–6. 10.2337/diabetes.54.3.751 15734852

[9] Summermatter S Marcelino H Arsenijevic D Buchala A Aprikian O Assimacopoulos-Jeannet F. Adipose tissue plasticity during catch-up fat driven by thrifty metabolism: relevance for muscle-adipose glucose redistribution during catch-up growth. Diabetes (2009) 58(10):2228–37. 10.2337/db08-1793 PMC2750217 19602538

[10] Marcelino H Veyrat-Durebex C Summermatter S Sarafian D Miles-Chan J Arsenijevic D. A role for adipose tissue de novo lipogenesis in glucose homeostasis during catch-up growth: a Randle cycle favoring fat storage. Diabetes (2013) 62(2):362–72. 10.2337/db12-0255 PMC3554390 22961086

[11] Crescenzo R Lionetti L Mollica MP Ferraro M D’Andrea E Mainieri D. Altered skeletal muscle subsarcolemmal mitochondrial compartment during catch-up fat after caloric restriction. Diabetes (2006) 55(8):2286–93. 10.2337/db06-0312 16873692

[12] Summermatter S Mainieri D Russell AP Seydoux J Montani JP Buchala A. Thrifty metabolism that favors fat storage after caloric restriction: a role for skeletal muscle phosphatidylinositol-3-kinase activity and AMP-activated protein kinase. FASEB J (2008) 22(3):774–85. 10.1096/fj.07-8972com 17928359

[13] De Andrade PB Neff LA Strosova MK Arsenijevic D Patthey-Vuadens O Scapozza L. Caloric restriction induces energy-sparing alterations in skeletal muscle contraction, fiber composition and local thyroid hormone metabolism that persist during catch-up fat upon refeeding. Front Physiol (2015) 6:254. 10.3389/fphys.2015.00254 26441673 PMC4584973

[14] Simonides WS van Hardeveld C. Thyroid hormone as a determinant of metabolic and contractile phenotype of skeletal muscle. Thyroid (2008) 18(2):205–16. 10.1089/thy.2007.0256 18279021

[15] Dentice M Marsili A Ambrosio R Guardiola O Sibilio A Paik JH. The FoxO3/type 2 deiodinase pathway is required for normal mouse myogenesis and muscle regeneration. J Clin Invest (2010) 120(11):4021–30. 10.1172/JCI43670 PMC2964991 20978344

[16] Salvatore D Simonides WS Dentice M Zavacki AM Larsen PR. Thyroid hormones and skeletal muscle–new insights and potential implications. Nat Rev Endocrinol (2014) 10(4):206–14. 10.1038/nrendo.2013.238 PMC4037849 24322650

[17] Calonne J Isacco L Miles-Chan J Arsenijevic D Montani JP Guillet C. Reduced Skeletal Muscle Protein Turnover and Thyroid Hormone Metabolism in Adaptive Thermogenesis That Facilitates Body Fat Recovery During Weight Regain. Front Endocrinol (Lausanne) (2019) 10:119. 10.3389/fendo.2019.00119 30873123 PMC6403129

[18] Mainieri D Summermatter S Seydoux J Montani JP Rusconi S Russell AP. A role for skeletal muscle stearoyl-CoA desaturase 1 in control of thermogenesis. FASEB J (2006) 20(10):1751–3. 10.1096/fj.06-5934fje 16809433

[19] Hill JO Anderson JC Lin D Yakubu F. Effects of meal frequency on energy utilization in rats. Am J Physiol (1988) 255:R616–21. 10.1152/ajpregu.1988.255.4.R616 3177693

[20] Calonne J Arsenijevic D Scerri I Miles-Chan JL Montani JP Dulloo AG. Low 24-hour core body temperature as a thrifty metabolic trait driving catch-up fat during weight regain after caloric restriction. Am J Physiol Endocrinol Metab (2019b) 317(4):E699–709. 10.1152/ajpendo.00092.2019 31430205

[21] Friedrichsen S Christ S Heuer H Schafer MK Mansouri A Bauer K. Regulation of iodothyronine deiodinases in the Pax8-/- mouse model of congenital hypothyroidism. Endocrinology (2003) 144:777–84. 10.1210/en.2002-220715 12586753

[22] Kester MH Toussaint MJ Punt CA Matondo R Aarnio AM Darras VM. Large induction of type III deiodinase expression after partial hepatectomy in the regenerating mouse and rat liver. Endocrinology (2009) 150:540–5. 10.1210/en.2008-0344 18787028

[23] Silvestri E Burrone L de Lange P Lombardi A Farina P Chambery A. Thyroid-state influence on protein-expression profile of rat skeletal muscle. J Proteome Res (2007) 6(8):3187–96. 10.1021/pr0701299 17608400

[24] Salzano AM Novi G Arioli S Corona S Mora D Scaloni A. Mono-dimensional blue native-PAGE and bi-dimensional blue native/urea-PAGE or/SDS-PAGE combined with nLC-ESI-LIT-MS/MS unveil membrane protein heteromeric and homomeric complexes in Streptococcus thermophilus. J Proteomics (2013) 94:240–61. 10.1016/j.jprot.2013.09.007 24061001

[25] Shinoda K Tomita M Ishihama Y. emPAI Calc--for the estimation of protein abundance from large-scale identification data by liquid chromatography-tandem mass spectrometry. Bioinformatics (2010) 26(4):576–7. 10.1093/bioinformatics/btp700 20031975

[26] Bianchi M Giacomini E Crinelli R Radici L Carloni E Magnani M. Dynamic transcription of ubiquitin genes under basal and stressful conditions and new insights into the multiple UBC transcript variants. Gene (2015) 573(1):100–9. 10.1016/j.gene.2015.07.030 26172870

[27] Ruiz-Alcaraz AJ Lipina C Petrie JR Murphy MJ Morris AD Sutherland C. Obesity-induced insulin resistance in human skeletal muscle is characterised by defective activation of p42/p44 MAP kinase. PloS One (2013) 8(2):e56928. 10.1371/journal.pone.0056928 23468892 PMC3585240

[28] Chen SN Czernuszewicz G Tan Y Lombardi R Jin J Willerson JT. Human molecular genetic and functional studies identify TRIM63, encoding Muscle RING Finger Protein 1, as a novel gene for human hypertrophic cardiomyopathy. Circ Res (2012) 111(7):907–19. 10.1161/CIRCRESAHA.112.270207 PMC3482312 22821932

[29] Araujo RL Andrade BM da Silva ML Ferreira AC Carvalho DP. Tissue-specific deiodinase regulation during food restriction and low replacement dose of leptin in rats. Am J Physiol Endocrinol Metab (2009) 296(5):E1157–63. 10.1152/ajpendo.90869.2008 19208852

[30] Araujo RL Carvalho DP. Bioenergetic impact of tissue-specific regulation of iodothyronine deiodinases during nutritional imbalance. J Bioenerg Biomembr (2011) 43(1):59–65. 10.1007/s10863-011-9327-x 21249435

[31] Beukhof CM Medici M van den Beld AW Hollenbach B Hoeg A Visser WE. Selenium Status Is Positively Associated with Bone Mineral Density in Healthy Aging European Men. PloS One (2016) 11(4):e0152748. 10.1371/journal.pone.0152748 27055238 PMC4824523

[32] Peeters RP Wouters PJ Kaptein E van Toor H Visser TJ Van den Berghe G. Reduced activation and increased inactivation of thyroid hormone in tissues of critically ill patients. J Clin Endocrinol Metab (2003) 88(7):3202–11. 10.1210/jc.2002-022013 12843166

[33] McAninch EA Bianco AC. Thyroid hormone signaling in energy homeostasis and energy metabolism. Ann N Y Acad Sci (2014) 1311:77–87. 10.1111/nyas.12374 24697152 PMC4451242

[34] Bianco AC Salvatore D Gereben B Berry MJ Larsen PR. Biochemistry, cellular and molecular biology, and physiological roles of the iodothyronine selenodeiodinases. Endocr Rev (2002) 23(1):38–89. 10.1210/edrv.23.1.0455 11844744

[35] Araujo RL de Andrade BM de Figueiredo AS da Silva ML Marassi MP Pereira Vdos S. Low replacement doses of thyroxine during food restriction restores type 1 deiodinase activity in rats and promotes body protein loss. J Endocrinol (2008) 198(1):119–25. 10.1677/JOE-08-0125 18430765

[36] Giacco A Delli Paoli G Simiele R Caterino M Ruoppolo M Bloch W. Exercise with food withdrawal at thermoneutrality impacts fuel use, the microbiome, AMPK phosphorylation, muscle fibers, and thyroid hormone levels in rats. Physiol Rep (2020) 8(3):e14354. 10.14814/phy2.14354 32034884 PMC7007447

[37] de Vries EM van Beeren HC van Wijk ACWA Kalsbeek A Romijn JA Fliers E. Regulation of type 3 deiodinase in rodent liver and adipose tissue during fasting. Endocr Connect (2020) 9(6):552–62. 10.1530/EC-20-0189 PMC7354722 32449699

[38] Wiles CM Young A Jones DA Edwards RH. Muscle relaxation rate, fibre-type composition and energy turnover in hyper- and hypo-thyroid patients. Clin Sci (Lond) (1979) 57(4):375–84. 10.1042/cs0570375 509876

[39] Johnson SM Connelly S Fearns C Powers ET Kelly JW. The transthyretin amyloidoses: From delineating the molecular mechanism of aggregation linked to pathology to a regulatory-agency-approved drug. J Mol Biol (2012) 421:185–203. 10.1016/j.jmb.2011.12.060 22244854 PMC3350832

[40] Richardson SJ. Cell and molecular biology of transthyretin and thyroid hormones. Int Rev Cytol (2007) 258:137–93. 10.1016/S0074-7696(07)58003-4 17338921

[41] Lee EJ Bhat AR Kamli MR Pokharel S Chun T Lee YH. Transthyretin is a key regulator of myoblast differentiation. PloS One (2013) 8:e63627. 10.1371/journal.pone.0063627 23717457 PMC3661549

[42] Alshehri B D’Souza DG Lee JY Petratos S Richardson SJ. The diversity of mechanisms influenced by transthyretin in eurobiology: Development, disease and endocrine disruption. J Neuroendocrinol (2015) 27:303–23. 10.1111/jne.12271 25737004

[43] Lee EJ Shaikh S Choi D Ahmad K Baig MH Lim JH. Transthyretin Maintains Muscle Homeostasis Through the Novel Shuttle Pathway of Thyroid Hormones During Myoblast Differentiation. Cells (2019) 8(12):1565. 10.3390/cells8121565 PMC6952784 31817149

[44] Morkin E. Control of cardiac myosin heavy chain gene expression. Microsc Res Tech (2000) 50(6):522–31. 10.1002/1097-0029(20000915)50:6<522::AID-JEMT9>3.0.CO;2-U 10998641

[45] Wiesner RJ Kurowski TT Zak R. Regulation by thyroid hormone of nuclear and mitochondrial genes encoding subunits of cytochrome-c oxidase in rat liver and skeletal muscle. Mol Endocrinol (1992) 6(9):1458–67. 10.1210/mend.6.9.1331777 1331777

[46] Zhang H Wang Y Li J Yu J Pu J Li L. Proteome of skeletal muscle lipid droplet reveals association with mitochondria and apolipoprotein a-I. J Proteome Res (2011) 10(10):4757–68. 10.1021/pr200553c 21870882

[47] Pacifici F Arriga R Sorice GP Capuani B Scioli MG Pastore D. Peroxiredoxin 6, a novel player in the pathogenesis of diabetes. Diabetes (2014) 63(10):3210–20. 10.2337/db14-0144 24947358

[48] Finley D Ozkaynak E Varshavsky A. The yeast polyubiquitin gene is essential for resistance to high temperatures, starvation, and other stresses. Cell (1987) 48(6):1035–46. 10.1016/0092-8674(87)90711-2 3030556

[49] Fornace AJ Jr Alamo I Hollander MC Lamoreaux E. Ubiquitin mRNA is a major stress-induced transcript in mammalian cells. Nucleic Acids Res (1989) 17(3):1215–30. 10.1093/nar/17.3.1215 PMC331738 2537950

[50] Finch JS St John T Krieg P Bonham K Smith HT Fried VA. Overexpression of three ubiquitin genes in mouse epidermal tumors is associated with enhanced cellular proliferation and stress. Cell Growth Differ (1992) 3(5):269–78. 1321656

[51] Vihervaara A Sergelius C Vasara J Blom MA Elsing AN Roos-Mattjus P. Transcriptional response to stress in the dynamic chromatin environment of cycling and mitotic cells. Proc Natl Acad Sci USA (2013) 110(36):E3388–97. 10.1073/pnas.1305275110 PMC3767495 23959860

[52] Bianchi M Crinelli R Arbore V Magnani M. Induction of ubiquitin C (UBC) gene transcription is mediated by HSF1: role of proteotoxic and oxidative stress. FEBS Open Bio (2018) 8(9):1471–85. 10.1002/2211-5463.12484 PMC6120222 30186748

[53] Setsuie R Suzuki M Kabuta T Fujita H Miura S Ichihara N. Ubiquitin C-terminal hydrolase-L3-knockout miceare resistant to diet-induced obesity and show increased activation of AMP-activated protein kinase in skeletal muscle. FASEB J (2009) 23(12):4148–57. 10.1096/fj.09-132217 19671667

[54] Setsuie R Suzuki M Tsuchiya Y Wada K. Skeletal muscles of Uchl3 knockout mice show polyubiquitinated protein accumulation and stress responses. Neurochem Int (2010) 56(8):911–8. 10.1016/j.neuint.2010.03.021 20380862

[55] Schultze SM Hemmings BA Niessen M Tschopp O. PI3K/AKT, MAPK and AMPK signalling: protein kinases in glucose homeostasis. Expert Rev Mol Med (2012) 14:e1. 10.1017/S1462399411002109 22233681

[56] Henriksen EJ Diamond-Stanic MK Marchionne EM. Oxidative stress and the etiology of insulin resistance and type 2 diabetes. Free Radic Biol Med (2011) 51(5):993–9. 10.1016/j.freeradbiomed.2010.12.005 PMC3071882 21163347

[57] Diamond-Stanic MK Marchionne EM Teachey MK Durazo DE Kim JS Henriksen EJ. Critical role of the transient activation of p38 MAPK in the etiology of skeletal muscle insulin resistance induced by low-level in vitro oxidant stress. Biochem Biophys Res Commun (2011) 405(3):439–44. 10.1016/j.bbrc.2011.01.049 PMC3042539 21241662

[58] Al-Lahham R Deford JH Papaconstantinou J. Mitochondrial-generated ROS down regulates insulin signaling via activation of the p38MAPK stress response pathway. Mol Cell Endocrinol (2016) 419:1–11. 10.1016/j.mce.2015.09.013 26454089

[59] Bodine SC Latres E Baumhueter S Lai VK Nunez L Clarke BA. Identification of ubiquitin ligases required for skeletal muscle atrophy. Science (2001) 294(5547):1704–8. 10.1126/science.1065874 11679633

