# Supplementary figures and images for: Adaptive Thermogenesis Driving Catch-Up Fat Is Associated With Increased Muscle Type 3 and Decreased Hepatic Type 1 Iodothyronine Deiodinase Activities: A Functional and Proteomic Study
Source: Front Endocrinol (Lausanne). 2021 Mar 4;12:631176. doi: 10.3389/fendo.2021.631176 (PMC7971177; doi:10.3389/fendo.2021.631176)

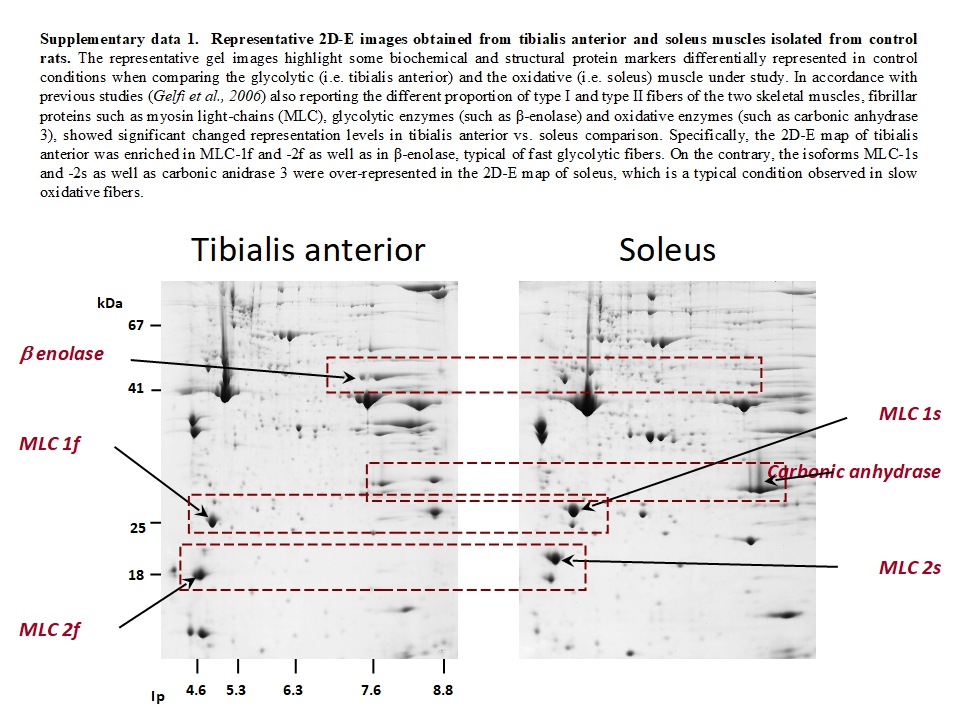

Supplement: Supplementary file 1 [file Image_1.jpeg]
